# Polyethylene Glycol Nanocolloids as Advanced Phase Change Materials for Sustainable Energy: Experimental Data on Viscosity, Density, and Isobaric Heat Capacity

**DOI:** 10.3390/polym18060673

**Published:** 2026-03-10

**Authors:** Cătălin Andrei Ţugui, Nicoleta Cojocariu, Bogdan Pricop, Dana Bejan, Alina Adriana Minea

**Affiliations:** 1 Faculty of Materials Science and Engineering, Technical University “Gheorghe Asachi” of Iasi, 700050 Iasi, Romania; catalin-andrei.tugui@academic.tuiasi.ro (C.A.Ţ.); nicoleta.cojocariu@student.tuiasi.ro (N.C.); bogdan.pricop@academic.tuiasi.ro (B.P.); 2 “Petru Poni” Institute of Macromolecular Chemistry, 700487 Iasi, Romania; bejan.dana@icmpp.ro

**Keywords:** PEG, MWCNT nanoparticles, density, viscosity, isobaric heat capacity

## Abstract

Polyethylene glycols (PEGs) are emerging as superior and accessible phase change materials and heat transfer fluids, offering improved thermal properties over conventional thermal oils to meet the demand for innovative, sustainable energy solutions. While general research on PEG performance is still scarce, this paper contributes relevant experimental data. As part of a broad investigation into PEG and PEG-based nanocolloids, this experiment helps to clarify the true potential of these new fluids by outlining both their key advantages and their operational limitations. Consequently, PEG 200 and two PEG 200 + PEG 400 mixtures were considered as base fluids for manufacturing MWCNT nanocolloids, resulting in 15 samples that were thoroughly investigated in terms of density, viscosity and isobaric heat capacity variation with both nanoparticle concentration and temperature. Results revealed that nanocolloid density follows the basic rules for nanoparticle-enhanced fluids, with moderate increase with nanoparticle addition and temperature. Viscosity increased with MWCNT concentration and decreased with temperature, while isobaric heat capacity upsurges with nanoparticle addition. These findings are critical, as they can shed some light into the practical benefits, while clearly explaining the potential drawbacks, of employing these novel fluids in heat transfer applications.

## 1. Introduction

Nanocolloids refer to nanoparticle-enhanced fluids, a term coined over 20 years ago as nanofluids. In recent decades, research on these fluids has focused on both experimental and numerical studies; however, most numerical approaches remain limited due to inconsistencies in evaluating thermophysical properties. On the other hand, experimental research has demonstrated a strong need for new heat transfer fluids and reliable databases of their properties. The following section presents the state-of-the-art in line with this paper’s aim and scope—specifically focusing on polyethylene glycols (PEGs) and carbon nanotube (CNT) nanoparticles (NPs).

CNTs are preferred for nanocolloid formulations due to their high thermal conductivity, but they significantly affect fluid viscosity, especially when forming percolation networks. Viscosity increases with both concentration and aspect ratio, with single-walled CNTs (SWCNTs) exhibiting stronger effects than MWCNTs (multi-walled carbon nanotubes) [1]. CNTs display quantum behavior in isobaric heat capacity at low temperatures, whilst SWCNTs display linear temperature dependence [1,2]. The thermal conductivity of individual SWCNTs and MWCNTs can exceed 2000 W/m·K and 3000 W/m·K, respectively, while CNT networks show lower effective values due to inter-tube resistance [1]. Surface modifications such as β-cyclodextrin functionalization improve dispersion and thermal conductivity with minimal increases in viscosity [3].

Cabaleiro et al. [4] presented the preparation and characterization of phase change materials (PCMs) based on PEG 400, enhanced with various nanopowders such as carbon black and mixtures of graphite/diamond. The nanoparticle concentrations ranged from 0.5 wt.% to 1 wt.%, and the suspensions were obtained using a two-step method. First, the powders were weighed on an analytical balance with an accuracy of 1 × 10^−4^ g, followed by mechanical shaking in a vortex mixer for 30 min. Finally, the suspensions were sonicated for 200 min in an ultrasonic bath, while periodically replacing the bath water to prevent overheating. The measured dynamic viscosity of the suspensions exhibited an average deviation of 2.9% compared to the values reported by Marcos et al. [5] for PEGs of similar molecular mass. The suspensions demonstrated non-Newtonian, shear-thinning behavior that became more pronounced with increasing particle content. Thermal conductivity measurements were performed using a THW-L2 (transient hot wire) device based on the transient hot-wire technique, with a declared accuracy of 5%. The thermal conductivity enhancement ranged from 2% to 3.6%, lower than the 12.7% increase reported by Marcos et al. [6] for PEG 400-based nanofluids containing 1 wt.% MWCNTs. Density was determined experimentally using a DMA 500 densimeter at atmospheric pressure and temperatures between 288.15 K and 313.15 K, with accuracies of ±0.02 K for temperature and ±0.0005 g/cm^3^ for density. Density values increased with nanoparticle concentration and were not significantly affected by temperature. A similar 0.42% increase was reported by Marcos et al. [6] for MWCNT (1 wt.%)/PEG 400 suspensions.

Marcos et al. [7] analyzed PEG 200 and PEG 300 base fluids combined with various concentrations of Baytubes^®^ C150P MWCNTs characterized by lengths of 10–20 μm, external diameters of 20–30 nm, internal diameters of 2–6 nm, specific surface area of 197 m^2^/g (BET method), and packed density of 1.8 g/cm^3^. The nanofluids were prepared via a two-step process involving weighing, mechanical stirring, and 30 min of sonication. Unlike pure PEG 200 and PEG 300, all resulting nanofluids exhibited pseudoplastic (shear-thinning) behavior, which became more pronounced with increasing MWCNT loading. Marcos et al. [6] reported shear-thinning viscosities for PEG 400 nanofluids containing up to 1 wt.% MWCNTs. Experimental viscosities measured at a shear rate of ≈800 s^−1^ showed significant increases—102% for PEG 200 and 71% for PEG 300—due to nanotube dispersion.

Naddaf et al. [8,9] investigated the performance of diesel oil enhanced with carbon-based nano-additives, including graphene nanoplatelets (GNPs) and MWCNTs, at weight concentrations of 0.05%, 0.1%, 0.2%, and 0.5%. They observed substantial improvements in both thermal and electrical conductivity, with the highest improvements occurring at 0.5 wt.%.

Rebrović et al. [10] examined how different types of MWCNTs affect the thermal and flow properties of PEG 200. Four types of MWCNT—differing in size and surface oxidation—were tested at concentrations up to 10 wt.%. The most significant thermal conductivity improvement (133%) was achieved with oxidized, short, and wide MWCNTs. Viscosity tests showed that PEG 2000 transitioned from Newtonian to shear-thinning behavior above 0.1 wt.% MWCNTs, with the effect intensifying at higher concentrations. Agglomeration was most pronounced with long, thin, pristine nanotubes. The experimental data aligned best with the Maxwell model for thermal conductivity and the Einstein model for viscosity, suggesting spherical clustering of nanotubes within the fluid.

Shoghl et al. [11] evaluated the thermophysical properties (i.e., electrical conductivity, density, and viscosity) of six water-based nanofluids across varying nanoparticle type, concentrations and temperatures. The addition of nanoparticles significantly increased electrical conductivity and viscosity. ZnO nanofluids exhibited the highest conductivity at low concentrations, while CNT nanofluids dominated at higher ones. A linear relationship between conductivity and concentration was observed for all nanofluids except MWCNTs, which exhibited a percolation threshold at 0.1 wt.%.

Živković et al. [12] measured the viscosity and density of PEG 200 and PEG 400 at temperatures ranging from 288.15 K to 333.15 K. They found that density decreases with increasing temperature, while viscosity decreases as well. PEG 400 mixtures displayed significantly higher viscosities than PEG 200 due to their longer polymer chains.

Mirahmad et al. [13] reviewed the dual role of MWCNTs in enhancing both thermal conductivity and specific heat capacity, while emphasizing the trade-offs with viscosity in nanofluid design. The authors noted that MWCNTs exhibit exceptional thermal conductivity (up to 3000 W/m·K). Their dispersion stability—achieved through sonication, surfactants, and pH control—is critical, as MWCNTs strongly influence thermophysical properties such as thermal conductivity, viscosity, and specific heat, often involving a trade-off between improved heat transfer and increased flow resistance.

Manimaran et al. [14] investigated MWCNT-based nanofluids at concentrations of 0.1–1.0 wt.%, prepared via two-step methods involving mechanical stirring and 30 min of ultrasonication. They demonstrated improved dispersion and stability when functionalized, leading to significant thermal conductivity enhancements (up to ~22% at 1 wt.%) while maintaining moderate viscosity increases and Newtonian behavior at low concentrations.

Razzaq et al. [15] also used MWCNTs as high-performance additives due to their exceptional thermal conductivity (up to 3000 W/m·K). Their covalent functionalization markedly improved dispersion stability, critical for long-term nanofluid performance. Moreover, hybrid nanofluids incorporating MWCNTs with other nanoparticles often exhibit superior thermal conductivity and stability compared to single-component systems, although optimization of surfactant type, pH, and ultrasonication remains essential to prevent aggregation and manage viscosity effects.

Okonkwo et al. [16] provided a comprehensive review of recent advancements in the preparation methods, thermophysical properties, and applications of nanofluids in various heat transfer systems, including solar collectors, heat exchangers, refrigeration units, radiators, thermal storage systems, and electronic cooling, especially focusing on carbon-based nanoparticles.

Das et al. [17] conducted an extensive review of the preparation methods, stabilization techniques, and thermophysical properties of single and hybrid nanofluids, focusing on their applications and challenges in heat transfer systems. Authors concluded that multi-walled carbon nanotubes (MWCNTs) are widely used in hybrid nanofluids due to their high thermal conductivity and large aspect ratio, which significantly enhance heat transfer performance when combined with metal or metal oxide nanoparticles.

### Relevance to SDG 7

Polyethylene glycols (PEGs) directly support the move toward sustainable development goal 7 (SDG 7)—affordable, reliable, and sustainable energy—primarily through their role in thermal energy storage (TES) systems for renewable energy infrastructure. A detailed breakdown of how PEG properties relate to key renewable energy applications is inserted as Table 1. The most significant application of PEGs in renewable energy is as a phase change material (PCM) in latent heat thermal energy storage (LHTES). This technology helps address the intermittency challenge of solar and other renewables, thereby increasing their reliability (a core component of SDG 7).

The intrinsic properties of PEGs make them one of the most sustainable and modern material choices (see Table 1).

However, while PEG is promising for a lot of applications, pure PEG has several limitations that current research aims to overcome: for example, its low thermal conductivity. This is a common issue for organic PCMs, slowing down the charging and discharging of heat. Researchers are overcoming this by creating nano-enhanced composites (e.g., adding carbon nanotubes or metal nanoparticles) to boost thermal transfer rates.

In essence, this study provides a material solution that enhances the environmental profile and technical performance necessary for achieving the global mandate of clean and accessible energy for everyone. More exactly, the aim of this research is to investigate the density, viscosity, and isobaric heat capacity of three types of suspensions consisting of polyethylene glycol (PEG) as the base fluid and multi-walled carbon nanotube (MWCNT) nanoparticles in concentrations ranging from 0.05 to 3 wt.%. The novelty of this study relies on the first-time employment of PEG mixtures as base fluids. The study followed a strict experimental protocol and employed state-of-the-art equipment, with measurement uncertainties carefully controlled during the entire experimental chain, being part of a complex investigation into the PEG impact on heat transfer. The following sections will detail the experimental procedures, characterization methods, and a comprehensive discussion of the findings and their impact.

## 2. Experimental

The compounds used in this study were obtained from Merck (Darmstadt, Germany): PEG 400 (Kollisolv^®^ PEG E 400, CAS No. 25322-68-3), PEG 200 (CAS No. 25322-68-3), and MWCNT (CAS No. 308068-56-6)—see Table 2. Nanocolloids were prepared using the two-step method, which involved dispersing nanoparticles into the base fluids followed by 20 min of mechanical stirring and 60 min of sonication to enhance stability and reduce sedimentation. Sonication was carried out using a Geti GUC02A ultrasonic bath (ultrasound power: 60 W; frequency: 40 Hz).

For this particular study, nanoparticles were dispersed in PEG 200 and two mixtures of PEG 200 and PEG 400 (i.e., F3: 0.50 PEG 200 + 0.50 PEG 400, F4: 0.25 PEG 200 + 0.75 PEG 400), with MWCNT mass concentrations ranging from 0.05 wt.% to 0.3 wt.%.

The MWCNT content was kept low to limit the increase in viscosity. The novel base fluids (F3, F4) and nanocolloids are listed in Table 3, and further details regarding the intrinsic properties of the F3 and F4 fluids can be found in Bejan et al. [18]. Bejan et al. [8] reported for the first time a complete study in terms of DSC, TG, thermal conductivity, effusivity, as well as other properties for a number of PEG mixtures and PEG mixtures with water. From this study, it was concluded that F3 and F4 have clear advantages for heat transfer applications. The concentration of the samples was calculated in terms of mass fraction (*x*), while the conversion between mass and volume fraction was:

(1) $$\frac{1}{\varphi }=1+\frac{\rho_{p}}{\rho_{bf}}\left(\frac{1-x}{x}\right)$$

The conversion was applied to compare the experimental data with theoretical models available for density, viscosity, and isobaric heat capacity.

All experiments were carried out according to strict protocol designed to ensure reliability of results. This protocol encompassed all stages of the experimental process, including sample preparation (mixture and nanocolloid fabrication) and thermophysical property measurements. The experimental setup and methodology are summarized in Table 4.

The stability of the suspensions was evaluated through pH measurements using an Edge Multiparameter HI 2030 device (Hanna Instruments, Cluj Napoca, Romania).

**Figure 1:**
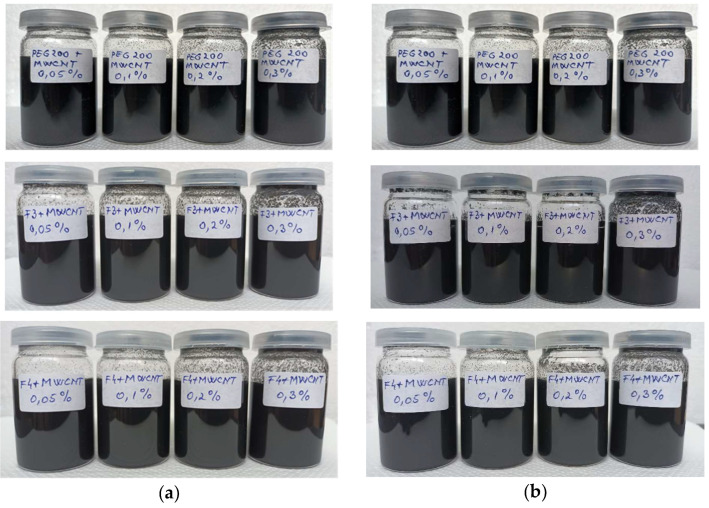
Photos of samples: (**a**) after two days, (**b**) after 20 days.

The MWCNT nanoparticles’ morphology was thoroughly investigated via SEM, TEM, PXRD, and porosity measurements (results available in Chereches et al. [19]). Sample preparation strictly adhered to the lab’s established protocol [18,19,20], which involved precise steps like calculation, weighting, mixing, and homogenization. Crucially, the suspensions underwent ultrasonic homogenization for 60 min. This specific duration was chosen based on the research team’s extensive background in creating stable nanocolloids (further insights are provided in [19,20]). Suspension stability was monitored for several days using visual inspection and pH readings (ranging from 7.77 to 8.4), confirming the nanocolloids’ stability was acceptable (prepared samples are shown in Figure 1). More details about pH measurements are largely discussed in Cojocariu et al. [21].

## 3. Experimental Results and Discussion

### 3.1. Thermal Analysis

**Figure 2:**
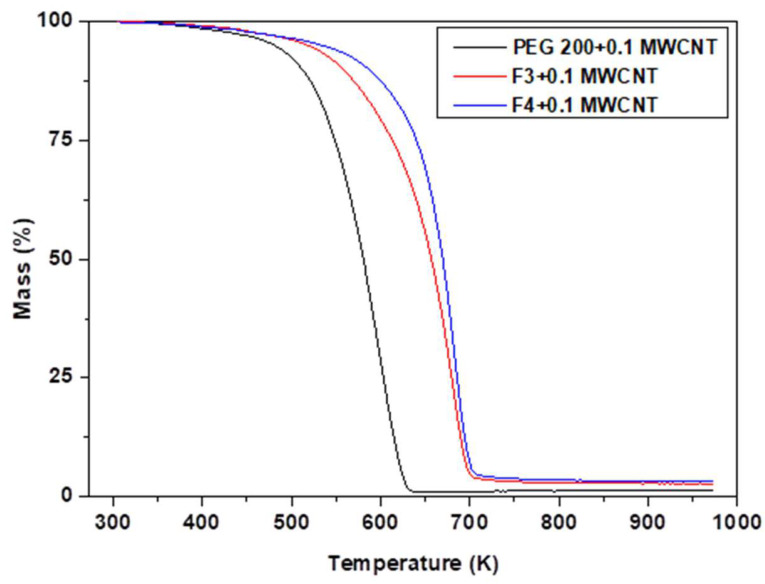
TGA thermograms of nanocolloids: PEG 200, F3, and F4 with 0.1% MWCNT.

**Figure 3:**
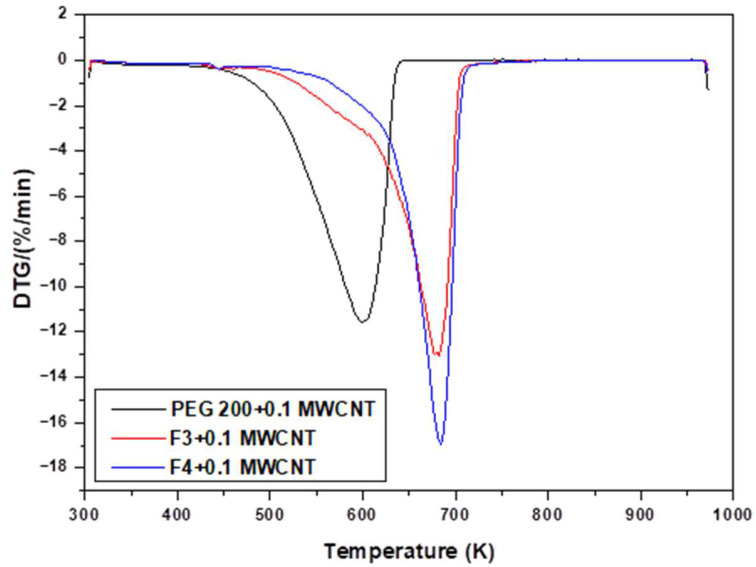
DTG for nanocolloids: PEG 200, F3, and F4 with 0.1% MWCNT.

Thermal analysis, in terms of TGA (thermogravimetric analysis), was performed for several samples with MWCNT in order to check their thermal stability, as well as total mass loss (see Table 5 and Figure 2 and Figure 3).

The decomposition of the F3 and F4 mixtures occurs in one degradation step, as was reported in the previous studies performed by Bejan et al. [18].

Figure 2 and Figure 3 depict the TG and DTG curves of the nanocolloids recorded at heating rates of 10 K/min between 303.15 and 973.15 K. As can be observed from the curves, the samples with 0.1 wt.%. MWCNT nanoparticles were relatively stable, with high weight loss over 627 K for PEG 200 + 0.1 MWCNT, 696 K for F3 + 0.1 MWCNT, and 697 K for F4 + 0.1 MWCNT, respectively (see Table 5). By comparing the temperature trend and the DTG/DTA peaks previously reported in the pure fluids (see Bejan et al. [18]) with those obtained for the nanocolloids presented in this study (see Table 5), a clear increase of a few degrees (up to 6 K) can be observed in the case of these samples. This thermal shift clearly indicates that the presence of nanoparticles contributes to an enhancement in the thermal stability of the systems.

Furthermore, the PEG 200 + 0.1 MWCNT nanocolloid exhibits lower thermal stability than F3 + 0.1 MWCNT and F4 + 0.1 MWCNT, as evidenced by the sharp weight loss at 599.15 K observed in the DTG curve (black line) in Figure 3. The total weight loss of each sample in response to temperature is listed in Table 5 as well as the amount of the sample that was analyzed.

### 3.2. Density Results and Discussion

**Figure 4:**
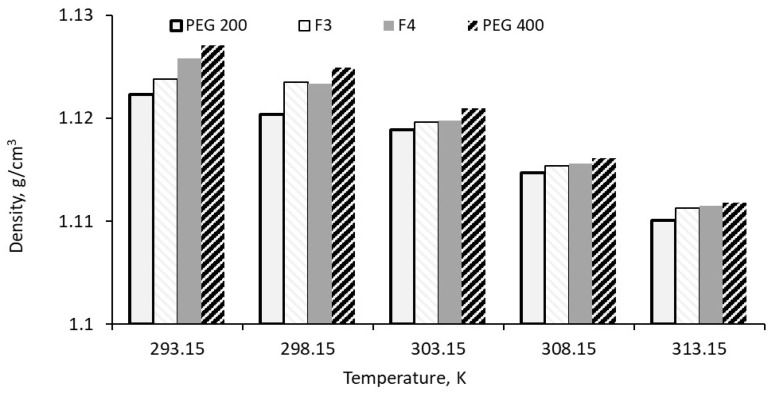
Base fluids’ density variation with temperature.

The densities of the mixtures and PEG 200 were measured at temperatures up to 313.15 K, with the results shown in Figure 4. Each experimental value represents the average of five measurements taken after cleaning the equipment according to the manufacturer’s recommendations. As observed in Figure 4, the density decreases with increasing temperature. This behavior is typical for all fluids, as heating raises their internal energy, causing atoms or molecules to move more rapidly and spread further apart.

**Figure 5:**
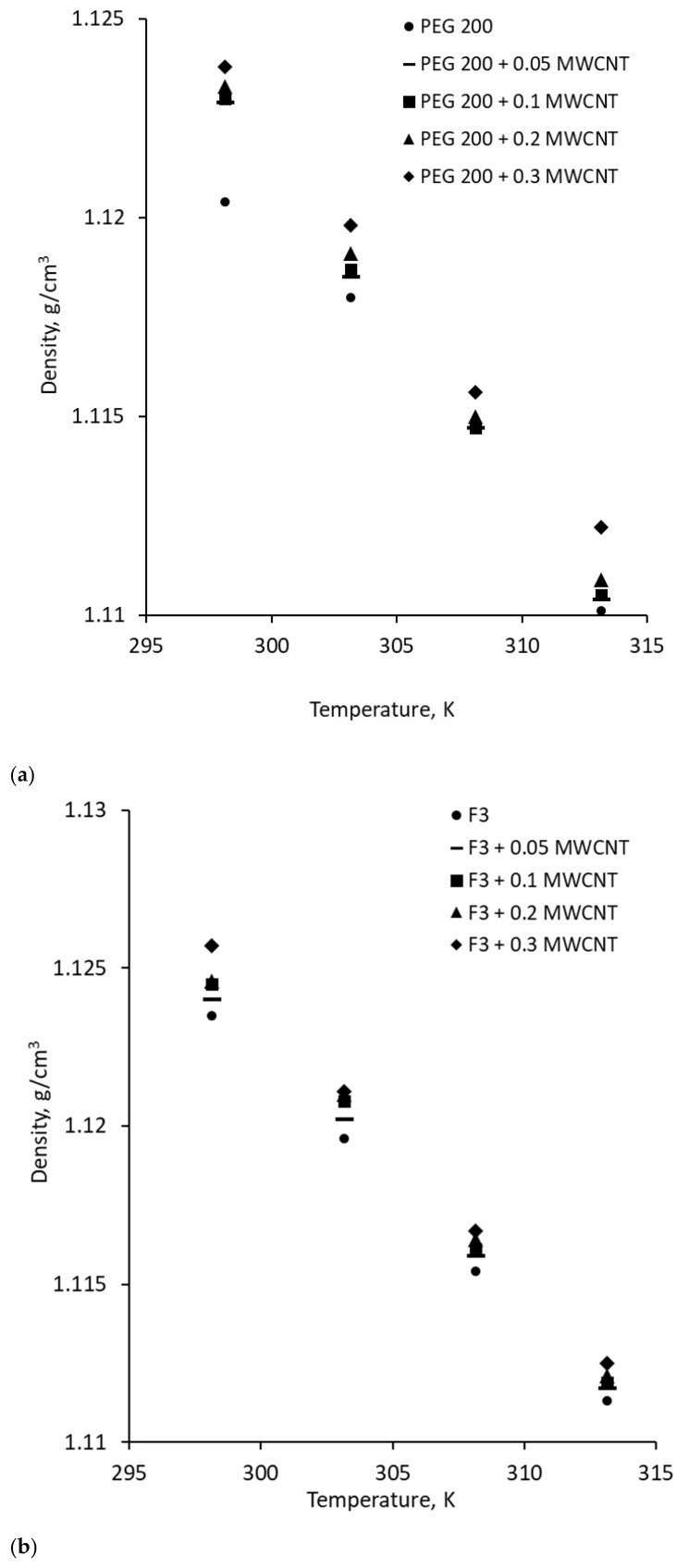

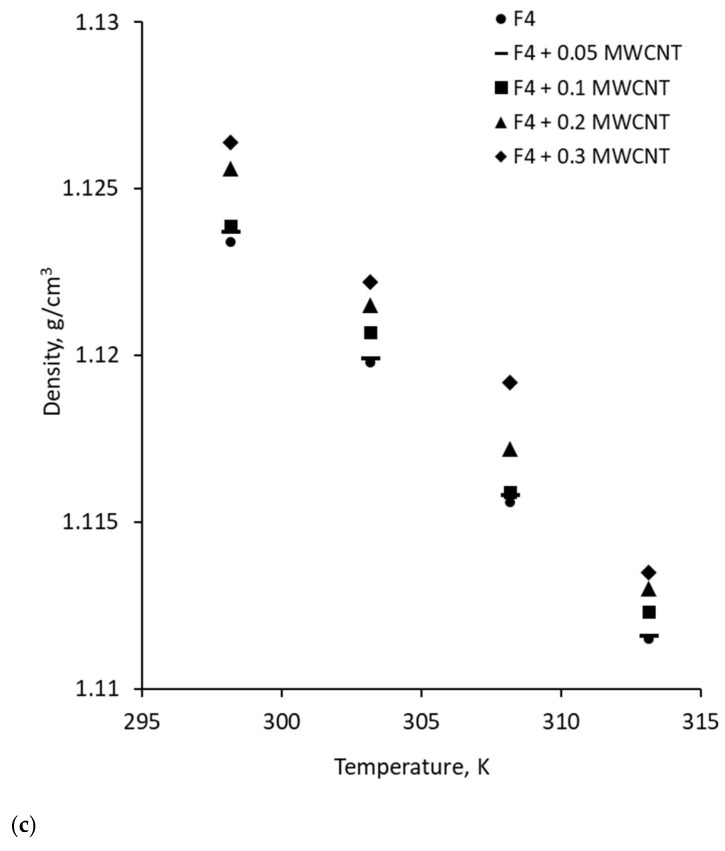
Density variation with temperature for all samples: (**a**) PEG 200 + MWCNT; (**b**) F3 + MWCNT; (**c**) F4 + MWCNT.

The density behavior of the nanocolloids under heating is graphically represented by the experimental values shown in Figure 5. At ambient temperature, as shown in Figure 5, the density increases with the addition of nanoparticles up to 0.5% for the suspension containing 0.3 wt.% MWCNT. This behavior is expected, as incorporating high-density solid nanoparticles into PEG raises the overall liquid density. Conversely, heating causes a typical decrease in density due to two effects: at the molecular level, heating increases the kinetic energy of molecules, causing them to move faster and spread farther apart; at the macroscopic level, the same mass occupies a larger volume, leading to a reduction in density (since density = mass/volume). The observed decrease in density is minor—up to 1.2% for all samples within the studied temperature range.

The data presented in Figure 5 were fitted using a simple linear equation expressed as:

*ρ* = a*T* + b (2)

In Equation (2), *ρ* is the density, *T* is temperature, while a and b are correlation coefficients that vary upon each type of suspension (see Table 6 for coefficients calculated for each sample).

The experimental values of the density for all samples were compared with the Pak and Cho [22] theoretical equation employed for most of the nanocolloids, that writes:

(3) $$\rho_{nf}=\rho_{np}\phi +\rho_{bf}(1-\phi )$$

where $\rho_{nf}$ is the nanocolloid density, $\rho_{np}$ is the nanoparticle density, $\rho_{bf}$ is the base fluid density, and ϕ is the nanoparticle volume fraction.

Comparing the data, a deviation of up to 0.208% was found (see Table 7 for details), proving that the Pak and Cho equation can be successfully used for the estimation of MWCNT nanocolloids. The deviation was calculated as the ratio between the experimental and theoretical value.

### 3.3. Viscosity Results and Discussion

The samples were tested at variable shear rates from 6.59 to 264 1/s. The results showed that at shear rates larger than 13.19 1/s, the torque exceeded 90%, meaning that the measurements were out of range, according to manufacturer indications. Thus, as recommended by the manufacturer, in order to maintain a measurement deviation below 1%, the experimental data were consequently collected at a shear rate of 10.56 1/s, corresponding to an RPM value of eight, to ensure a torque value between 10 and 90% regardless of the increasing temperature.

The viscosity of the samples was measured during heating up to 333.15 K, using small sample volumes of 6.7 mL, employing the VOLS-1 adapter from IKA (see the full description in Section 2).

The accuracy of viscosity measurements was verified by comparing the viscosities of PEG 200 and PEG 400 with data from the manufacturer and the literature. A deviation of approximately 3% was observed compared to the results reported by Cabaleiro et al. [4] and Chereches et al. [19,20]. It is worth noting that these studies employed different equipment, which explains the observed deviation.

**Figure 6:**
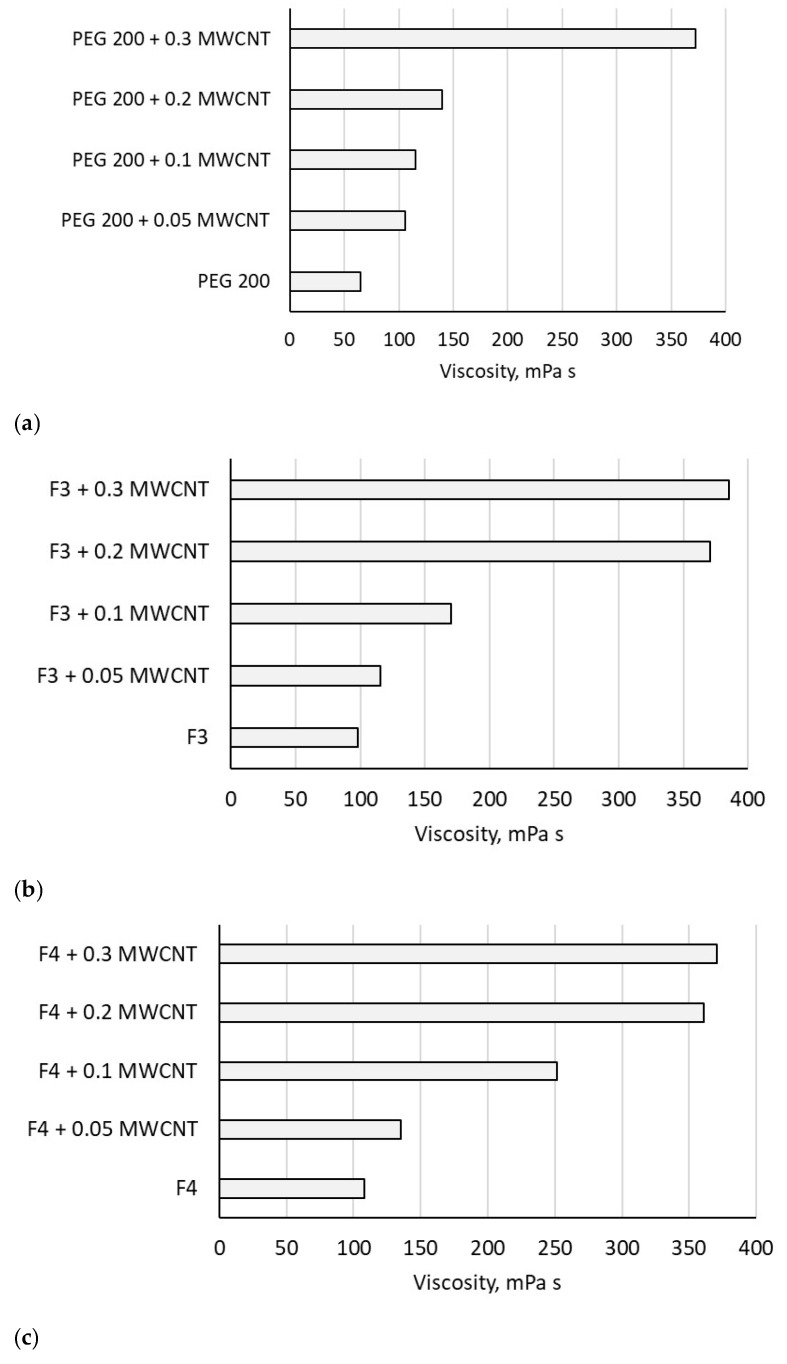
Influence of MWCNTs on viscosity of PEG at 293.15 K: (**a**) PEG 200 nanocolloids; (**b**) F3 nanocolloids; (**c**) F4 nanocolloids.

**Figure 7:**
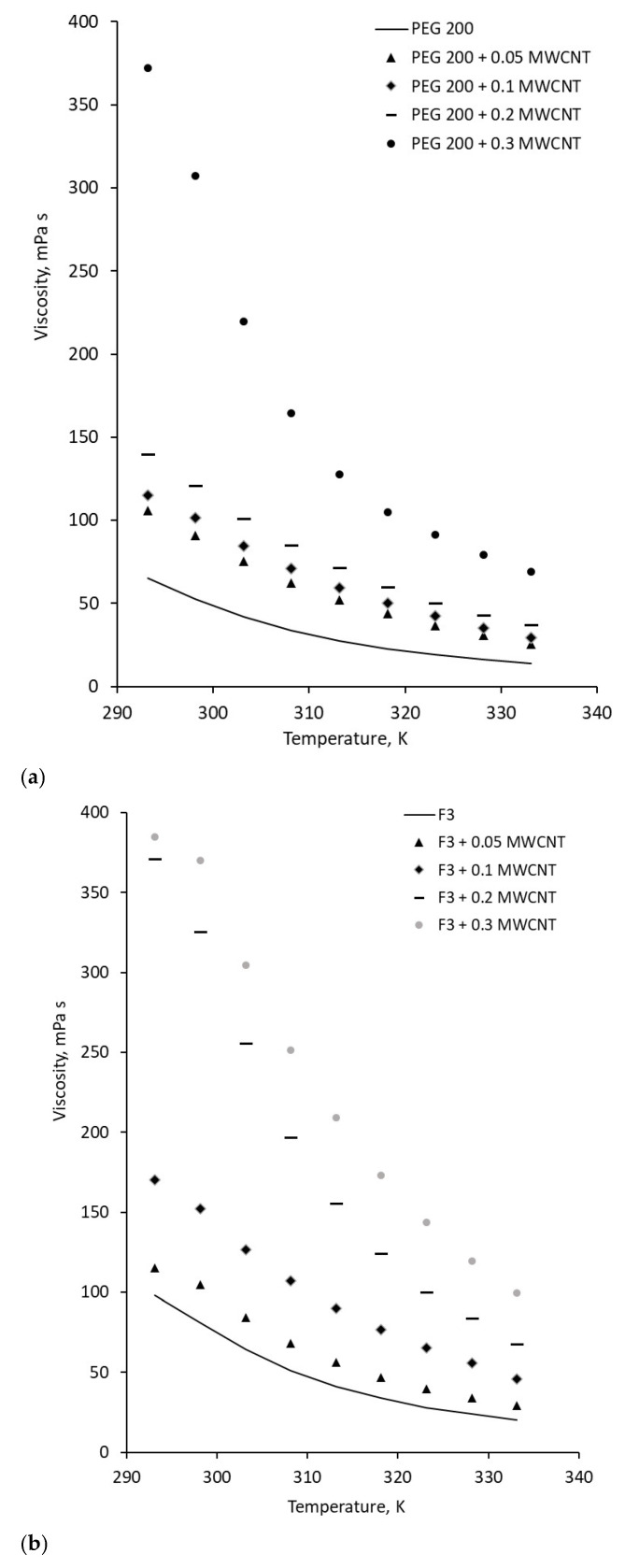

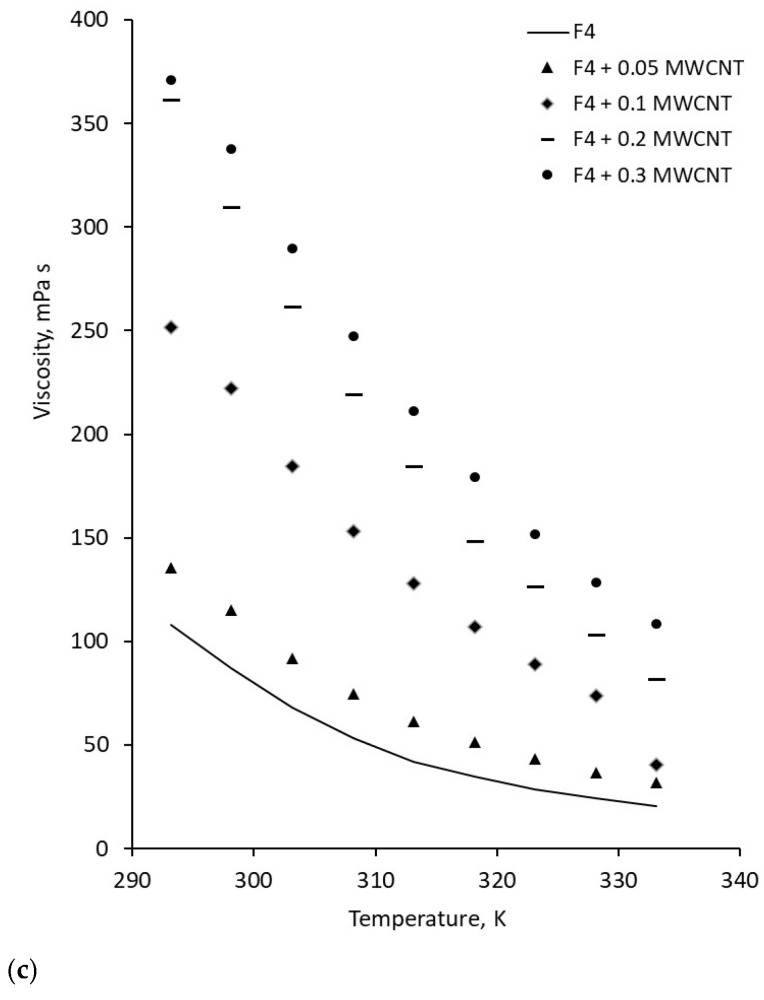
Temperature influence on viscosity samples at 8 RPM: (**a**) PEG 200 nanocolloids; (**b**) F3 nanocolloids; (**c**) F4 nanocolloids.

The viscosity of the F3 sample was 98.3 mPa·s, while PEG 200 and F4 exhibited viscosities of 65 mPa·s and 108 mPa·s, respectively. The addition of MWCNTs to the fluids led to an increase in viscosity at ambient temperature, as shown in Figure 6, whereas Figure 7 illustrates the temperature-dependent viscosity variation for all samples.

Viscosity increases with MWCNT addition, which is a normal phenomenon when solid nanoparticles are added to a liquid (see Figure 7). The increase is high even for low-loaded suspensions; it reaches 391.66% for 0.3 wt.% MWCNT in F3, proving that the addition of MWCNT has a big impact on nanocolloid viscosity regardless of concentration.

The heating of samples generates a decrease in viscosity, following normal fluid behavior as can be seen from Figure 7. The same trend was also noticed in the open literature, as well as from our group’s previous investigations (see refs. [4,19,20]).

The experimental data are compared in terms of volume fraction, with equations available in the open literature (see Table 8), and the results are shown in Table 9. The conversion between mass fraction and volume fraction (φ) was achieved with Equation (1).

Data from Table 9 outlines the major difference between theoretical estimations and experimental values, proving that for MWCNT nanocolloids, it is mandatory that the viscosity is experimentally checked and that no correlation can predict its increase, which is the same situation for most of the other tested nanocolloids.

**Figure 8:**
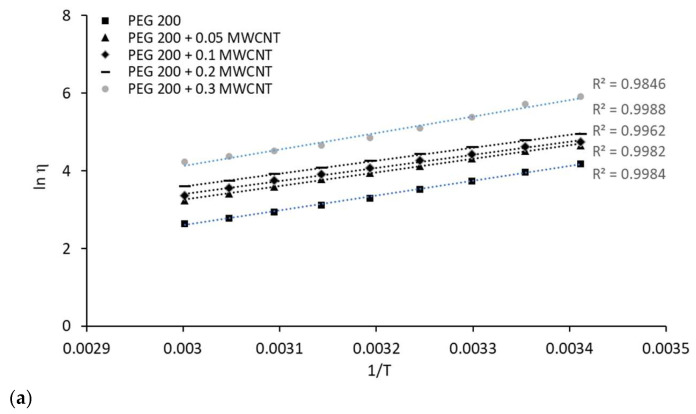

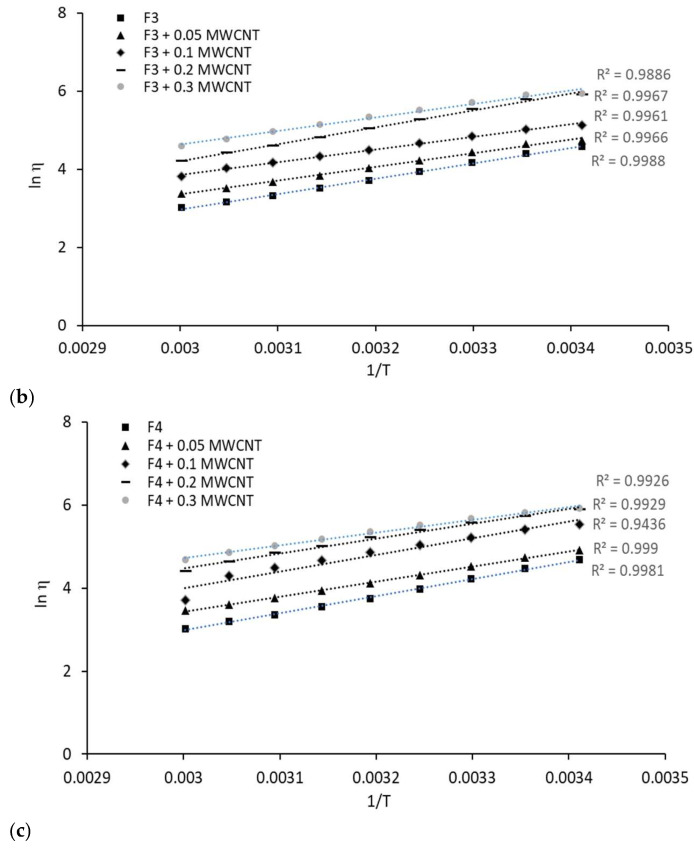
Experimental data plotted against Arrhenius fitting: (**a**) PEG 200 nanocolloids; (**b**) F3 nanocolloids; (**c**) F4 nanocolloids.

To study how dynamic viscosity (*η*) changed with temperature, we fit the experimental data using both the Arrhenius and a non-Arrhenius model. The Arrhenius-type equation used for viscosity is a direct analogy to the formula originally developed for reaction rates:

(4) $$\ln\eta \left(T\right)=\ln A+\frac{E_{a}}{R}\cdot \frac{1}{T}$$

where *η* is the dynamic viscosity, *T* is temperature, *E*_a_ is the energy activation for viscous flow and *R* is the universal gas constant. Although the original equation was created by Arrhenius [27] for chemical reactions, its application to viscosity is commonly credited to Guzman [28] and later popularized by Andrade [29]. We noted that if a liquid is measured across a very narrow temperature range, the plot of ln *η* versus 1/*T* can appear linear, resulting in a statistically strong fit (high *R*-squared values) for the simple Arrhenius equation. The corresponding ln *η* versus 1/*T* data for all samples were plotted to evaluate the Arrhenius fit, and the results are presented in Figure 8.

While the simple Arrhenius model may be adequate for practical engineering or quick data correlation, it is essentially an empirical simplification. Nevertheless, it is crucial not to rely on the Arrhenius model to draw conclusions about the fundamental physical properties of the liquid or to extrapolate viscosity data far beyond the measured temperature range. Its perceived accuracy in a small range does not reflect the true physical behavior of the liquid across a broad domain.

For a more accurate description, wider applicability, and better extrapolation over a large temperature range, a non-Arrhenius model like the Vogel–Fulcher–Tammann (VFT) equation is a superior choice (as detailed in references [30,31,32]). The VFT model is:

(5) $$\ln\eta \left(T\right)=\ln\eta_{0}+\frac{A\;T_{0}}{{T-T}_{0}}$$

where *η*_0_ is the value of viscosity when temperature tends to infinite and represents the energy associated with “cage” confinement due to close packing of liquid molecules, *A* is Angell strength and *T*_0_ is Vogel temperature. The experimental outcomes were fitted against these three parameters for every nanocolloid and the results are presented in Table 10.

Concluding, the Vogel–Fulcher–Tammann (VFT) equation is a highly effective and commonly used model for viscosity, and it provides an excellent fit for our nanocolloid samples. The model’s success is confirmed by high accuracy (R-squared value very close to 1) and low standard errors. The viscosity correlations found in Table 10 are valid under the following specific conditions: nanocolloids containing up to 0.3 wt.% MWCNT suspended in a PEG 200 or F3/F4 mixture in the interval of 293.15–333.15 K.

Additionally, a more complex data fitting was performed using an extension of the VFT equation in the attempt to capture both temperature and concentration influence. The employed equation is:

(6) $$\eta \left(T,\phi_{vol}\right)=\eta_{0}\cdot e^{\frac{AT_{0}}{T-T_{0}}}+B\cdot e^{\frac{B}{T}}\phi_{vol}+C\cdot \phi_{vol}^{2}$$

where *A*, *B* and *C* values are outlined in Table 11 for all nanocolloids.

#### Viscosity Hysteresis

The viscosity hysteresis phenomenon plays a crucial role in practical heat transfer systems, particularly in applications such as heat exchangers, where fluid stability and consistency directly influence performance. To evaluate this behavior, the samples were subjected to multiple heating–cooling cycles to assess the effect of MWCNT incorporation on each PEG-based fluid. This effect was first reported by Nguyen et al. [33] and has since been examined in several studies by different research groups [4,5,6,7,19,20].

**Figure 9:**
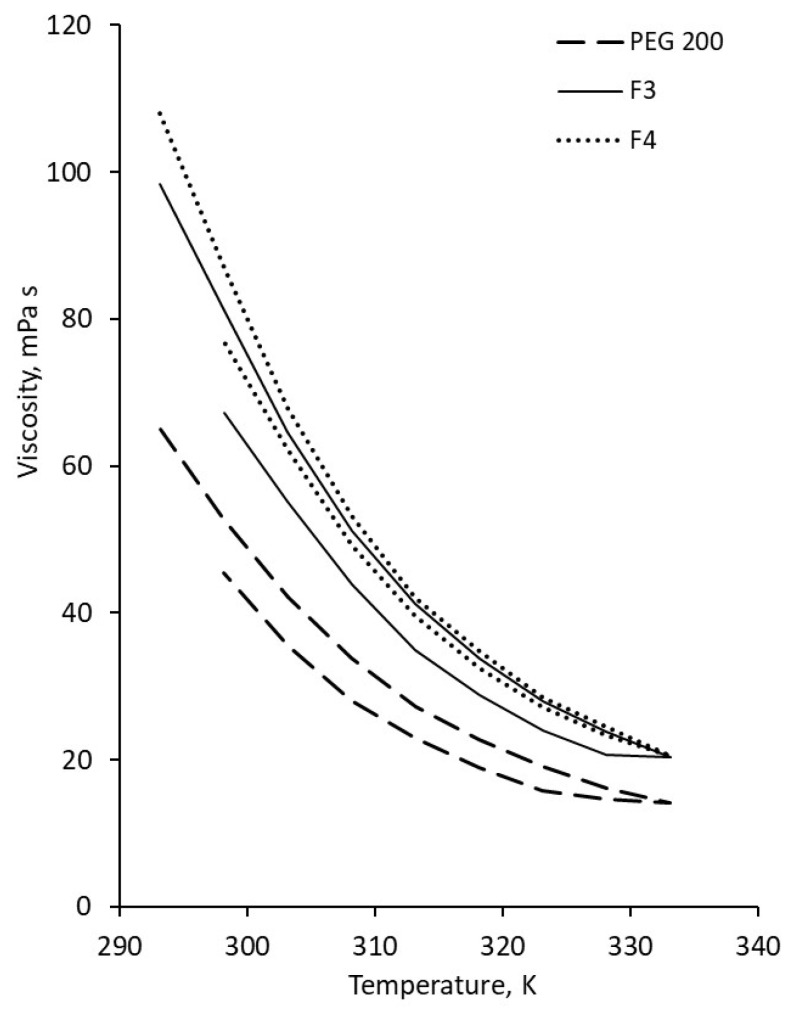
Viscosity hysteresis for PEG-based fluids.

The experimental results confirm that PEG mixtures exhibit viscosity hysteresis, which progressively decreases after two consecutive heating–cooling cycles (see Figure 9).

A comparable trend was observed across all noncolloidal systems, indicating that the addition of MWCNTs promotes the upsurge in hysteresis (see Table 12). This increase may be attributed to the formation of microstructural interactions between MWCNTs and PEG chains, which hinder molecular mobility during thermal cycling. Such interactions could lead to energy dissipation through structural rearrangements, thereby amplifying the hysteretic behavior observed in the nanocolloids [33].

This phenomenon arises from the temporary breakdown and incomplete recovery of the fluid’s internal molecular structure. Heating increases molecular kinetic energy, accelerating movement and weakening intermolecular forces, which immediately decreases viscosity as documented in prior studies (see refs. [3,4,5]). Upon cooling, molecules return to a lower energy state but often fail to completely restore their original, more entangled or structured arrangement. This incomplete structural recovery, especially across multiple thermal cycles, leads to a new, less organized state with reduced internal friction, allowing molecules to slide past each other more easily. This effect is particularly prominent in complex fluids like PEG (polyethylene glycol) mixtures, where shorter polymer chains, such as in PEG 200, are inherently more mobile and less entangled, making them more susceptible to this permanent structural thinning. Additionally, adding small amounts of MWCNT nanoparticles tends to exacerbate this behavior due to reduced internal friction, a phenomenon that can be beneficial for heat transfer.

Moreover, in the PEG/MWCNT system, the viscosity percolation threshold is defined as the critical concentration where the carbon nanotubes stop behaving as isolated particles and begin to form a continuous, interconnected network that restricts the flow of the PEG polymer chains. For PEG/MWCNT nanocolloids used in thermal energy storage, this threshold was calculated as 0.19% based on experimental data for PEG 200 nanocolloids, 0.11% for F3 nanocolloids, and 0.04% for F4. The percolation threshold depends on temperature (i.e., as temperature increases, PEG viscosity drops, but the MWCNT network often remains stable, meaning the relative impact of the nanotubes on flow becomes even more pronounced), PEG molecular weight (i.e., higher molecular weight PEG has longer chains that entangle more easily with nanotubes, often lowering the threshold, explaining why PEG 200 has a higher threshold compared to F3 and F4 mixtures), as well as on MWCNT aspect ratio (i.e., the longer the nanotubes, the lower the concentration needed to “tangle” into a network).

### 3.4. Isobaric Heat Capacity Results and Discussion

**Figure 10:**
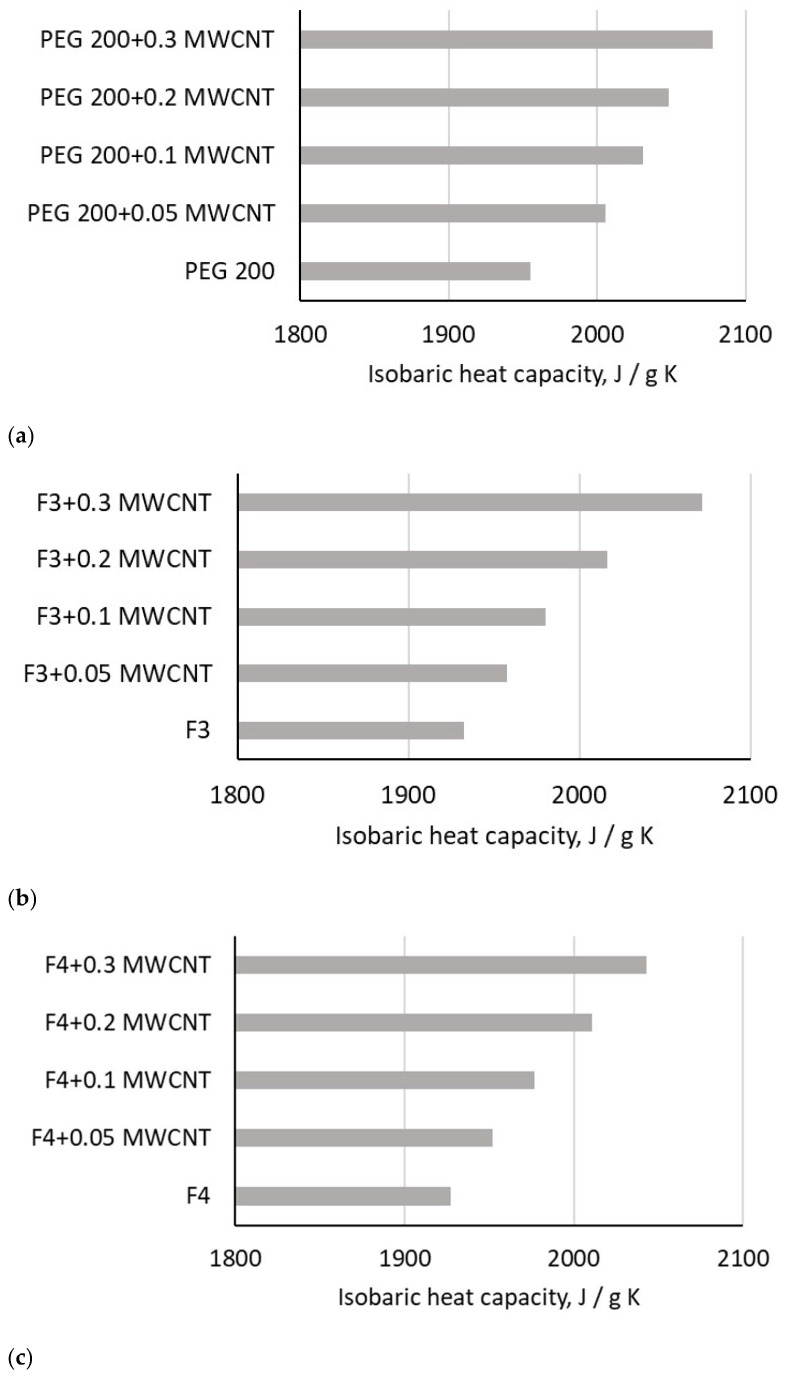
Experimental results on isobaric heat capacity at 293.15 K: (**a**) PEG 200 nanocolloids; (**b**) F3 nanocolloids; (**c**) F4 nanocolloids.

**Figure 11:**
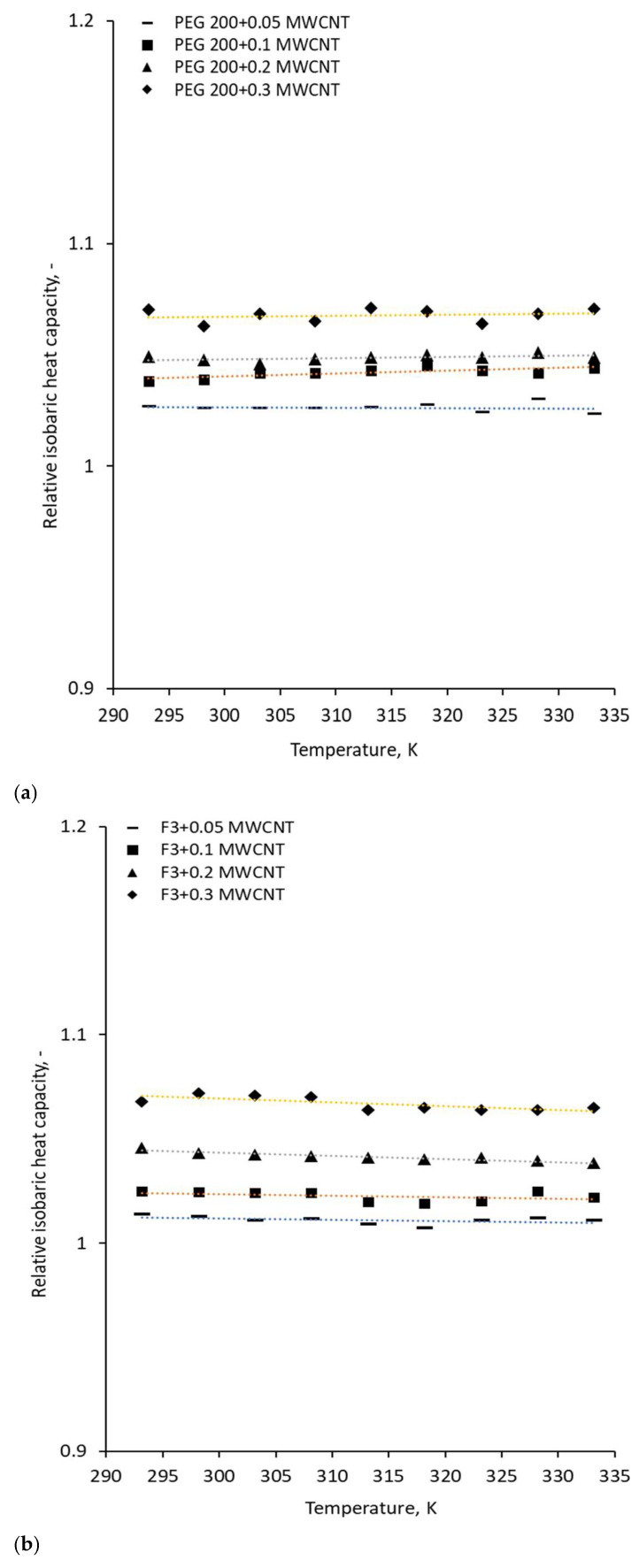

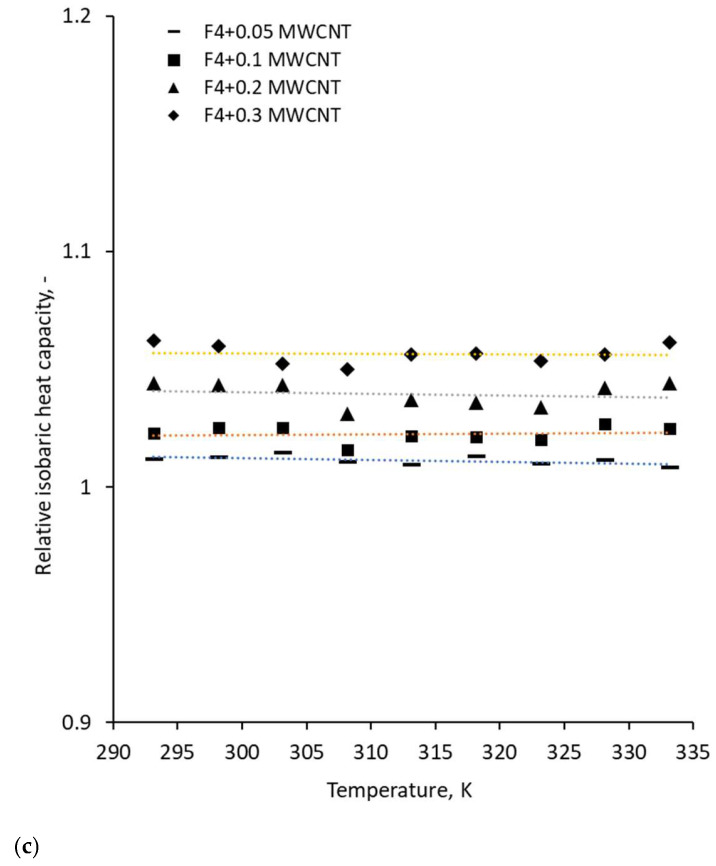
Temperature influence on isobaric heat capacity: (**a**) PEG 200 nanocolloids; (**b**) F3 nanocolloids; (**c**) F4 nanocolloids.

The experimental data were collected on C-Therm equipment (C-Therm, Fredericton, Canada) software, as was detailed in Section 2, and are presented in Figure 10 and Figure 11 for ambient temperature and temperature variation, respectively. By integrating C-Therm’s measurements (thermal conductivity and effusivity) with the experimental density data, the isobaric heat capacity was calculated directly from the equation:

(7) $$c_{p}=\;\frac{e^{2}}{k\rho }$$

where *k*, *ρ*, *e* and *c*_p_ are thermal conductivity, density, thermal effusivity and isobaric heat capacity, respectively. The thermal conductivity of MWCNT suspension in PEG 200 and PEG mixtures was previously reported (see Cojocariu et al. [34] for detailed insights), and the experimental data interpretation led to the conclusion that the addition of MWCNT increases the thermal conductivity of the samples, while the temperature influence is minimal.

Figure 10 shows that the addition of MWCNT nanoparticles leads to a linear enhancement of the PEG fluid’s isobaric heat capacity at ambient conditions. The degree of enhancement, which reached a maximum of 7.2% relative to the base fluid, shows a significant dependence on the MWCNT mass fraction. Given the lack of existing literature, these results are a novel contribution, strongly establishing a linear correlation between the relative isobaric heat capacity (ratio between the isobaric heat capacity of the nanocolloid and base fluid) and the mass fraction (w). This observed linearity is precisely captured by a fitting equation with an R-squared value approaching 0.99, as:

(8) $$\mathrm{for}\;\mathrm{PEG}\;200\;\mathrm{nanocolloids}\text{:}\;c_{pr}=18.663\;w+1.0109,\;R^{2}=0.89$$

(9) $$\mathrm{for}\;F3\;\mathrm{nanocolloids}\text{:}\;c_{pr}=23.294\;w+1.0003,\;R^{2}=0.99$$

(10) $$\mathrm{for}\;F4\;\mathrm{nanocolloids}\text{:}\;c_{pr}=19.675\;w+1.0027,\;R^{2}=0.99$$

Figure 11 demonstrates that the influence of the temperature increase on the isobaric heat capacity of all samples is irrelevant, the same behavior also being noticed for the thermal conductivity [34,35].

Concluding, the employment of PEGs as heat transfer media represents an emerging field, and a comprehensive understanding of their performance characteristics still remains incomplete. It is theorized that the variations in the isobaric specific heat capacity of PEG-based nanocolloids are predominantly influenced by two mechanisms: the formation of interfacial nanolayers at the solid–liquid interface and the intrinsic isobaric heat capacity of the nanoparticles. Consequently, rigorous, systematic investigations are essential to accurately ascertain the contributions of these mechanisms and facilitate the development of robust predictive models.

## 4. Conclusions

Several low molecular mass polyethylene glycols were involved in this experimental study of their properties. Density, viscosity and isobaric heat capacity results were here discussed in terms of nanoparticle concentration and temperature influence. The base fluids were PEG 200 and two PEG 200 + PEG 400 mixtures, which is a novel approach. MWCNTs were added to these base fluids, and the major findings can be synthesized as:

- The PEG mixtures, identified as F3 and F4, are novel fluids that were not explored beforehand.
- The density of nanocolloids with MWCNT follow the classical equations for nanoparticle-enhanced fluids. The density at ambient temperature moderately increases with addition of MWCNT.
- The variation in density with temperature of all samples was found to be linear and equations are projected.
- Viscosity of all nanocolloids greatly increases with MWCNT concentration, which is a normal phenomenon also found in the open literature.
- A novel approach is the discussion on viscosity variation with temperature and its comparison with classical equations for nanocolloids, as well as Arrhenius and non-Arrhenius approaches.
- Another novelty refers to the modified VFT equation for viscosity estimation with both temperature and nanoparticle fraction.
- At ambient temperature, the isobaric heat capacity of all nanocolloids linearly increases with nanoparticle loading, while temperature seems to have little to no effect in the studied range.

SDG 7 requires accessible, reliable, and modern energy, a goal supported by material innovation. PEGs are emerging as a new class of superior, accessible heat transfer fluids, but systematic data is scarce. This specific work, part of an extensive study on various PEG and PEG nanocolloid formulations, is therefore essential. It is designed to validate the performance benefits of PEGs and highlight any associated implementation hurdles, generating the critical data needed to deploy these promising materials in sustainable energy technologies.

## Figures and Tables

**Table 1:** Relevance to SDG 7.

PEG Property	Relevance to SDG 7
Renewable basis	PEGs can be synthesized from biomass-derived resources, reducing reliance on fossil fuels and directly supporting the “sustainable” element of the goal.
Low toxicity and non-corrosiveness	PEGs are generally non-toxic and chemically inert. This is a major advantage over corrosive or hazardous materials often used in heat transfer fluids, making the energy systems safer and environmentally benign.
High thermal stability	Their high thermal stability (over 573.15 K) means they can operate reliably at the medium temperatures relevant to many modern industrial heat recovery and concentrated solar power (CSP) applications without breaking down. This ensures long-term system reliability.
Cycling stability	PEGs demonstrate good chemical and thermal stability over thousands of charging/ discharging cycles, which is critical for the durability and longevity of energy storage devices.

**Table 2:** Properties of chemicals used, as per manufacturer data.

Chemical	Properties
PEG 400	CAS: 25322-68-3, Formula C_2_H_4_O Molecular weight: 380–420 g/mol Density: 1.125 g/cm^3^.
PEG 200	CAS: 25322-68-3, Formula (C_2_H_4_O)nH_2_O Molecular weight: 190–210 g/mol Density: 1.124 g/cm^3^
MWCNT	CAS Number: 308068–56-6 Dimensions: 50–90 nm diameter, 5–20 µm length, 88–100 walls Density: 2.100 g/cm^3^

**Table 3:** Samples.

Sample	PEG 200 Quantity, g	PEG 400 Quantity, g	MWCNT Quantity, g	Mass Concentration, %	Volume Concentration, %
F3: 0.50 PEG 200 + 0.50 PEG 400	3.7619	7.520	-	-	-
F4: 0.25 PEG 200 + 0.75 PEG 400	1.495	9.032	-	-	-
PEG 200 + 0.05 MWCNT	19.99	-	0.01	0.05	0.025
PEG 200 + 0.1 MWCNT	19.98	-	0.02	0.1	0.05
PEG 200 + 0.2 MWCNT	19.96	-	0.04	0.2	0.099
PEG 200 + 0.3 MWCNT	19.94	-	0.06	0.3	0.149
F3 + 0.05 MWCNT	6.663	13.327	0.01	0.05	0.025
F3 + 0.1 MWCNT	6.66	13.32	0.02	0.1	0.05
F3 + 0.2 MWCNT	6.653	13.307	0.04	0.2	0.099
F3 + 0.3 MWCNT	6.647	13.293	0.06	0.3	0.149
F4 + 0.05 MWCNT	2.856	17.134	0.01	0.05	0.025
F4 + 0.1 MWCNT	2.854	17.126	0.02	0.1	0.05
F4 + 0.2 MWCNT	2.851	17.109	0.04	0.2	0.099
F4 + 0.3 MWCNT	2.849	17.091	0.06	0.3	0.149

**Table 4:** Equipment and methodology.

Test Type	Equipment	Methodology
Preparation	KERN ADJ 100-4 (Kern, Balingen, Germany), uncertainty of 1 × 10^−3^ g for weighting, an ultrasonic homogenizer GETI GUC02A (TIPA, Opava, Czech Republic) with a power of 60 W and a frequency of 40 kHz.	The chemicals were weighed in calculated amounts to prepare each suspension. First, the PEG mixture was prepared, followed by the NP addition. Each suspension was then stabilized using a mechanical stirrer and an ultrasonic homogenizer for approximately one hour.
Viscosity	ROTAVISC (IKA, Staufen, Germany) lo-vi viscometer with a CBC VISC Lite system. Deviation: The manufacturer reported a viscosity uncertainty of 1% and repeatability of 0.2%. Temperature Control: Temperature was maintained within ±0.2 K, with stability of ±0.05 K according to DIN 12876. Adapter for low volumes VOLS-1, equipped with a double jacket with temperature sensor to quickly heat up or cool down the sample as well as a coaxial cylinder system for the analysis of the sample at a specific shear rate.	Viscosity measurements were performed between 298.15 K and 333.15 K in both heating and cooling modes, with a constant heating rate of 1 K/min, to assess thermal hysteresis (i.e., differences in properties during heating and cooling).
Density	Digital Densimeter DS7800 (Krus, Hamburg, Germany) Uncertainty: ±0.0001 g/cm^3^.	Density was determined between 298.15 K and 313.15 K.
Isobaric heat capacity	C-Therm Trident (C-Therm, Fredericton, Canada) Thermal Conductivity System with a Modified Transient Plane Source (MTPS) sensor. Vulcan furnace—for precise temperature control and a heating rate of up to 1 K/min to ensure steady-state measurements. Uncertainty: The system is reported to have a high accuracy, with an uncertainty of less than 1%.	Data were collected at ambient temperature up to 333.15 K. The isobaric heat capacity was determined based on the measured thermal effusivity, thermal conductivity, and density of each sample.
TGA analysis	STA 449 F1 JUPITER equipment (Netzsch, Waldkraiburg, Germany)	The tests were conducted in a nitrogen atmosphere using samples weighing 40 mg. The samples were heated in alumina crucibles with a heating rate of 10 K/min. Nitrogen was used as inert atmosphere at a flow rate of 50 mL/min. The experimental data were processed using the instrument software (NETZSCH PROTENS v. 4.2).

**Table 5:** TGA results for fluids (PEG 200, F3 and F4) with 0.1 wt.% MWCNT nanoparticles.

Sample	T Onset, K	T Peak (DTG), K	T Endset, K	Total Weight Loss at 972.65 K, %	Mass Probe, mg
PEG 200 + 0.1 MWCNT	519.85	599.15	626.95	1.25	40.28
F3 + 0.1 MWCNT	576.35	677.45 682.55	695.45	2.67	78.39
F4 + 0.1 MWCNT	585.15	683.75	697.25	3.14	85.42

**Table 6:** Coefficients and density deviation of the correlation.

Fluid	Coefficients		R-Squared Value
	a	b	a
PEG 200	−0.0006	1.1353	0.946
PEG 200 + 0.05 MWCNT	−0.0007	1.1389	0.966
PEG 200 + 0.1 MWCNT	−0.0007	1.1392	0.968
PEG 200 + 0.2 MWCNT	−0.0007	1.1393	0.965
PEG 200 + 0.3 MWCNT	−0.0007	1.1387	0.962
F3	−0.0007	1.1386	0.949
F3 + 0.05 MWCNT	−0.0007	1.1395	0.952
F3 + 0.1 MWCNT	−0.0007	1.1408	0.958
F3 + 0.2 MWCNT	−0.0007	1.1409	0.956
F3 + 0.3 MWCNT	−0.0008	1.1431	0.972
F4	−0.0007	1.1399	0.978
F4 + 0.05 MWCNT	−0.0007	1.1403	0.978
F4 + 0.1 MWCNT	−0.0008	1.1442	0.997
F4 + 0.2 MWCNT	−0.0008	1.1449	0.996
F4 + 0.3 MWCNT	−0.0008	1.1458	0.988

**Table 7:** Deviation between experimental and theoretical data at 273.15 K.

Fluid	Experimental Value	Theoretical Value	Deviation, %
PEG 200 + 0.05 MWCNT	1123.70	1122.54	−0.103
PEG 200 + 0.1 MWCNT	1123.90	1122.79	−0.099
PEG 200 + 0.2 MWCNT	1124.10	1123.27	−0.074
PEG 200 + 0.3 MWCNT	1124.40	1123.76	−0.057
F3 + 0.05 MWCNT	1124.50	1124.04	−0.041
F3 + 0.1 MWCNT	1125.30	1124.29	−0.090
F3 + 0.2 MWCNT	1125.40	1124.77	−0.056
F3 + 0.3 MWCNT	1126.80	1125.25	−0.137
F4 + 0.05 MWCNT	1125.20	1126.04	0.075
F4 + 0.1 MWCNT	1128.30	1126.29	−0.179
F4 + 0.2 MWCNT	1128.60	1126.76	−0.163
F4 + 0.3 MWCNT	1129.60	1127.25	−0.208

**Table 8:** Classic models for the theoretical estimation of nanocolloid viscosity.

Reference	Model	Comments
Einstein [23]	$\frac{\eta_{nf}}{\eta_{bf}}=1+2.5\;\varphi$	The equation is valid for low nanoparticle concentration.
Brinkman [24]	$\frac{\eta_{nf}}{\eta_{bf}}={\left(1-\varphi \right)}^{-2.5}$	
Maron–Pierce [25]	$\frac{\eta_{nf}}{\eta_{bf}}={\left(1-\frac{\varphi }{\varphi_{m}}\right)}^{-2}$	
Krieger–Dougherty [26]	$\frac{\eta_{nf}}{\eta_{bf}}={\left(1-\frac{\varphi }{\varphi_{m}}\right)}^{-\left[\eta \right]\varphi_{m}}$	[η] is the intrinsic viscosity ([η] = 2.5 for MWCNT nanoparticles), φ_m_ (φ_m_ ≈ 0.65) = maximum volume fraction.

**Table 9:** Relative viscosity: experimental versus theoretical results.

Volume Fraction	Experimental	Einstein Model	Brinkman Model	Krieger–Dougherty Model	Maron–Pierce Model
PEG 200 nanocolloids with MWCNT					
0.00025	1.721591	1.0020	1.0020	1.0020	1.0024
0.0005	1.918561	1.0040	1.0040	1.0040	1.0049
0.00099	2.280303	1.0079	1.0080	1.0080	1.0098
0.00149	5.816288	1.0200	1.0203	1.0204	1.0251
F3 nanocolloids with MWCNT					
0.00025	1.292232	1.0020	1.0020	1.0020	1.0024
0.0005	1.876695	1.0040	1.0040	1.0040	1.0049
0.00099	4.008631	1.0079	1.0080	1.0080	1.0098
0.00149	4.563502	1.0200	1.0203	1.0203	1.0251
F4 nanocolloids with MWCNT					
0.00025	1.32336	1.0020	1.0020	1.0020	1.0024
0.0005	2.554661	1.0040	1.0040	1.0040	1.0049
0.00099	3.559264	1.0079	1.0080	1.0080	1.0098
0.00149	3.887227	1.0200	1.0203	1.0204	1.0251

**Table 10:** Coefficients and statistics for standard VFT model.

Sample	T_0_, K	A	ln η_0_, mPa s	R-Squared Value	Standard Error of Viscosity
PEG 200	99.406	17.425	−4.767	0.99940	0.0198
PEG 200 + 0.05 MWCNT	28.40	101.299	−6.164	0.9989	0.0261
PEG 200 + 0.1 MWCNT	31.054	87.915	−5.615	0.9978	0.0359
PEG 200 + 0.2 MWCNT	31.859	84.234	−5.297	0.9999	0.0207
PEG 200 + 0.3 MWCNT	117.998	13.970	−3.513	0.9943	0.0738
F3	84.858	24.540	−5.391	0.9995	0.0190
F3 + 0.05 MWCNT	35.832	76.843	−05.894	0.9981	0.0342
F3 + 0.1 MWCNT	31.318	83.407	−04.784	0.9978	0.0343
F3 + 0.2 MWCNT	31.914	109.04	−07.332	0.9982	0.0413
F3 + 0.3 MWCNT	27.803	103.413	−4.768	0.9939	0.0619
F4	93.519	20.631	−4.939	0.9996	0.01801
F4 + 0.05 MWCNT	65.812	34.225	−04.978	0.9995	0.0172
F4 + 0.1 MWCNT	27.096	123.543	−06.927	0.9707	0.1608
F4 + 0.2 MWCNT	28.889	102.862	−5.291	0.9961	0.0516

**Table 11:** Coefficients and statistics for extended VFT model.

	Correlation Coefficient					R-Squared Value	Standard Error of Viscosity
	*η*_0_, mPa s	*A*	*T*_0_, K	*B*	*C*		
PEG 200	−4.65 × 10^1^	1.43 × 10^4^	1.44 × 10^4^	1.49 × 10^3^	−5.69 × 10^7^	0.84	41.08
F3	9.56 × 10^−8^	8.69 × 10^1^	5.63 × 10^1^	1.52 × 10^3^	−5.02 × 10^7^	0.97	25.51
F4	8.85 × 10^−6^	8.92 × 10^1^	4.52 × 10^1^	1.54 × 10^3^	−6.35 × 10^7^	0.98	17.61

**Table 12:** Hysteresis data for investigated samples.

Fluid	Viscosity Hysteresis, %	
	303.15 K	328.15 K
PEG 200	−18.87	−9.52
PEG 200 + 0.05 MWCNT	−32.33	34.90
PEG 200 + 0.1 MWCNT	−44.19	38.98
PEG 200 + 0.2 MWCNT	−111.13	51.70
PEG 200 + 0.3 MWCNT	−216.57	72.86
F3	−17.05	−14.97
F3 + 0.05 MWCNT	−21.12	17.34
F3 + 0.1 MWCNT	−59.37	45.55
F3 + 0.2 MWCNT	−104.80	61.70
F3 + 0.3 MWCNT	−108.48	72.55
F4	−9.16	−4.61
F4 + 0.05 MWCNT	−17.64	22.36
F4 + 0.1 MWCNT	−76.38	41.83
F4 + 0.2 MWCNT	−109.63	64.12
F4 + 0.3 MWCNT	−125.99	69.46

## Data Availability

The raw data supporting the conclusions of this article will be made available by the authors on request.

## Abbreviations

Nomenclature	
A	adjustable parameter in VFT equation, -
c_p_	isobaric heat capacity, J/kg K
e	thermal effusivity, W·s^1^/^2^/(m^2^·K)
E_a_	energy activation for viscous flows
R	universal gas constant
R^2^	R-squared value, -
s	standard deviation, -
T	temperature, K
T_0_	adjustable parameter in VFT equation, K
x	nanoparticle mass fraction, -
Greek symbols	
ϕ	nanoparticle volume concentration, %
[η]	intrinsic viscosity, -
η	dynamic viscosity, Pa s
η_0_	adjustable parameter in VFT equation, Pa s
ρ	density, kg/m^3^
φ	nanoparticle volume fraction, -
*φ_m_*	maximum volume fraction, -
Abbreviations	
CNT	carbon nanotubes
CSP	concentrated solar power
DTG	derivative thermogravimetric analysis
LHTES	latent heat thermal energy storage
MTPS	modified transient plane source
MWCNT	multi-walled carbon nanotube
NP	nanoparticles
PCM	phase change material
PEG	polyethylene glycol
SDG	Sustainable Development Goal
SWCNT	single wall carbon nanotubes
TES	thermal energy storage
TGA	thermogravimetric analysis
THW	transient hot wire
VFT	Vogel–Fulcher–Tammann
Subscripts:	
bf	refers to base fluid
exp	refers to experimental
m	refers to average values
nf	refers to nanocolloid
p	refers to particle
r	relative
vol	refers to volume
wt	refers to weight
